# Estimating Rare Disease Incidences With Large-scale Internet Search Data: Development and Evaluation of a Two-step Machine Learning Method

**DOI:** 10.2196/42721

**Published:** 2023-04-28

**Authors:** Jiayu Li, Zhiyu He, Min Zhang, Weizhi Ma, Ye Jin, Lei Zhang, Shuyang Zhang, Yiqun Liu, Shaoping Ma

**Affiliations:** 1 Department of Computer Science and Technology Tsinghua University Beijing China; 2 Institute for AI Industry Research Tsinghua University Beijing China; 3 Department of Medical Research Center Peking Union Medical College Hospital Beijing China; 4 Department of Nephrology Peking Union Medical College Hospital Beijing China; 5 Department of Cardiology Peking Union Medical College Hospital Beijing China

**Keywords:** disease incidence estimation, rare disease, internet search engine, infoveillance, deep learning, public health

## Abstract

**Background:**

As rare diseases (RDs) receive increasing attention, obtaining accurate RD incidence estimates has become an essential concern in public health. Since RDs are difficult to diagnose, include diverse types, and have scarce cases, traditional epidemiological methods are costly in RD registries. With the development of the internet, users have become accustomed to searching for disease-related information through search engines before seeking medical treatment. Therefore, online search data provide a new source for estimating RD incidences.

**Objective:**

The aim of this study was to estimate the incidences of multiple RDs in distinct regions of China with online search data.

**Methods:**

Our research scale included 15 RDs in China from 2016 to 2019. The online search data were obtained from Sogou, one of the top 3 commercial search engines in China. By matching to multilevel keywords related to 15 RDs during the 4 years, we retrieved keyword-matched RD-related queries. The queries used before and after the keyword-matched queries formed the basis of the RD-related search sessions. A two-step method was developed to estimate RD incidences with users’ intents conveyed by the sessions. In the first step, a combination of long short-term memory and multilayer perceptron algorithms was used to predict whether the intents of search sessions were RD-concerned, news-concerned, or others. The second step utilized a linear regression (LR) model to estimate the incidences of multiple RDs in distinct regions based on the RD- and news-concerned session numbers. For evaluation, the estimated incidences were compared with RD incidences collected from China’s national multicenter clinical database of RDs. The root mean square error (RMSE) and relative error rate (RER) were used as the evaluation metrics.

**Results:**

The RD-related online data included 2,749,257 queries and 1,769,986 sessions from 1,380,186 users from 2016 to 2019. The best LR model with sessions as the input estimated the RD incidences with an RMSE of 0.017 (95% CI 0.016-0.017) and an RER of 0.365 (95% CI 0.341-0.388). The best LR model with queries as input had an RMSE of 0.023 (95% CI 0.017-0.029) and an RER of 0.511 (95% CI 0.377-0.645). Compared with queries, using session intents achieved an error decrease of 28.57% in terms of the RER (*P*=.01). Analysis of different RDs and regions showed that session input was more suitable for estimating the incidences of most diseases (14 of 15 RDs). Moreover, examples focusing on two RDs showed that news-concerned session intents reflected news of an outbreak and helped correct the overestimation of incidences. Experiments on RD types further indicated that type had no significant influence on the RD estimation task.

**Conclusions:**

This work sheds light on a novel method for rapid estimation of RD incidences in the internet era, and demonstrates that search session intents were especially helpful for the estimation. The proposed two-step estimation method could be a valuable supplement to the traditional registry for understanding RDs, planning policies, and allocating medical resources. The utilization of search sessions in disease detection and estimation could be transferred to infoveillance of large-scale epidemics or chronic diseases.

## Introduction

### Background

Rare diseases (RDs) refer to a group of diseases with very low prevalence (usually less than 0.05% of the population [1]). There are more than 7000 known RDs and more than 400 million people are affected by RDs worldwide [2]. Because of their diseases, patients with RDs often experience social discrimination and financial hardship [3]. Most RDs have a genetic or congenital cause, and over half of patients with RDs have varying degrees of disabilities [4]. The burden of disease management and income decrease due to the disorders have resulted in poverty being a common experience for families coping with RDs [5]. Therefore, RDs have become an essential concern in public health, attracting substantial research attention.

Disease surveillance (ie, detecting the incidences of diseases) is a common but crucial method for understanding RDs [6]. Traditional surveillance registries are based on consistent case reporting from workers in ubiquitous surveillance systems [7]. However, RDs incidence detection is challenging for traditional registry systems for several reasons: (1) the diagnosis of most RDs is extremely complicated, and it takes approximately 6-8 years to get an accurate diagnosis [2], resulting in complex registry records of RD patients; (2) different RDs belong to different clinical departments or systems, making it difficult to integrate data from various registry institutions; and (3) the cases of RDs are so scarce that maintaining timely reports will be a resource-intensive task.

Therefore, researchers have been seeking to detect or estimate the incidences of RDs with indirect information. For instance, various international and national platforms were constructed for collecting RD knowledge and incidences [6,8,9].

With the development of the internet, a tremendous amount of data was created online. Infoveillance (ie, using online information for syndromic surveillance [10]) has been successfully applied in many studies [11]. Diverse sources of online data greatly enrich the information for disease estimation, such as Wikipedia views [12], News views [13], medical forum blogs [14], and search engine data [15].

Nevertheless, to our knowledge, no study has yet explored the possibility of using infoveillance data in RD incidences estimation, and the existing research has not paid attention to the context information of disease-related data in the online environment, such as searching sessions in the search engines. However, comparing online search data to RD incidences and further estimating RD incidences is beneficial. Search engine data will locate the patients and families from the source, which is more convenient than a multiround clinical diagnosis and registry. In addition, search engines provide unlimited information, which can be used to break the barriers between RDs in different clinical departments. Hence, search engine data can make it possible to estimate multiple RDs in multiple locations simultaneously.

### Prior Work

Because few studies have focused on estimating RD incidences with online information, we reviewed prior research about employing online data in detecting or estimating epidemic and chronic diseases, and evaluated their differences with respect to RD incidences estimation.

Since the spread of epidemic diseases will cause an increase of related online searches, several studies have focused on the detection and prediction of epidemic diseases using infoveillance methods [16]. The new approaches began with estimating trends of influenza [15,17]. Subsequently, the query volume of search engines has been widely used to detect flu [18,19], dengue [20], pandemic H1N1 [21], and other diseases. Beyond search data, Xu et al [22] further considered the influence of news, which was used to detect occurrences of hand-foot-and-mouth disease with related queries, news clicks, and page clicks, improving the disease detection performance. In recent years, geographical information has been considered for infoveillance. Researchers tried to predict flu trends in multiple locations simultaneously [19,23] or transferred a trained disease prediction model to new regions [24]. During the ongoing COVID-19 pandemic, web search data have also shown great utility in disease surveillance [25-27].

In addition to epidemics, infoveillance has also been utilized in chronic diseases and other disorders. Ram et al [28] tried to estimate the number of asthma patients at a specific hospital with data from Google Trends, Twitter, and nearby air quality. Correlation analysis between eye disease trends and related queries showed a significant interrelationship between disease cases and online data [29]. Tkachenko et al [30] revealed that Google Trends could detect early signs of diabetes by monitoring combinations of keywords in online search queries. Sleep disorders [31] and mental health problems [32] were also found to be related to search volumes.

These previous works on epidemics and chronic diseases showed great successes of infoveillance, which inspired us to apply search data for RDs incidence estimation. Nevertheless, existing methods cannot be used directly for RDs because RDs remarkably differ from epidemics or common chronic diseases. In all previous studies based on search engine data, disease-related queries were extracted and the number (volume) of queries was used as the model input. However, RD-related search behaviors may be caused by cyberchondria (ie, an unfounded escalation of anxiety about common symptomatology), as search engines can potentially escalate medical concerns [33]. Our experiment also revealed that RD-related search behaviors are sparse, and only a minority of them are actually based on a concern about RDs. Therefore, besides query numbers, more information related to users’ search process is needed for accurate RDs estimation.

### Objective

The aim of this study was to estimate the incidences of multiple RDs in distinct regions using search engine data.

As RD-related search behaviors are sparse and complex, it is not suitable to utilize RD-related query numbers directly for RD incidence estimation. Therefore, we designed a two-step machine learning method to estimate RD incidences with the volume of search sessions that concern RDs. The RD-related *queries* were selected by matching the search logs with RD-specific keywords. The *search sessions* were constructed with the queries submitted in the period before and after the RD-related queries.

The two-step method is as follows. In the first step, the intents of search sessions are predicted. Users’ search intents indicate their purpose when querying RD-related questions on the search engine. The intents vary when the users mention RD-related queries in the session, such as seeking medical resources for patients, learning about news, searching for answers to medical assignments, and out of curiosity. By identifying sessions specifically concerned with RDs, we could filter out the noise from the RD-related search data effectively. In the second step, the incidences of multiple RDs are estimated in multiple regions with the volume of different session intents. RD incidences could be estimated more accurately with the filtered session numbers. Following previous works on disease detection with search engine data [15,23,24,34], linear regression (LR) without autoregressive modeling of historical RD incidences was considered when estimating RD incidences from search session intents.

The novel aspects of this study are two-fold. First, to our best knowledge, this is the first study to utilize search engine data in the estimation of multiple RD incidences, paving a new direction for improved understanding of RDs. This study therefore provides a helpful supplement to traditional RD registry systems. Second, the proposed approach introduces search sessions, especially session intents, into search engine–based infoveillance. The experimental results showed significant improvement when session intents were considered. The search session information could also be applied for the infoveillance of other diseases.

## Methods

### Overview and Framework

**Figure 1:**
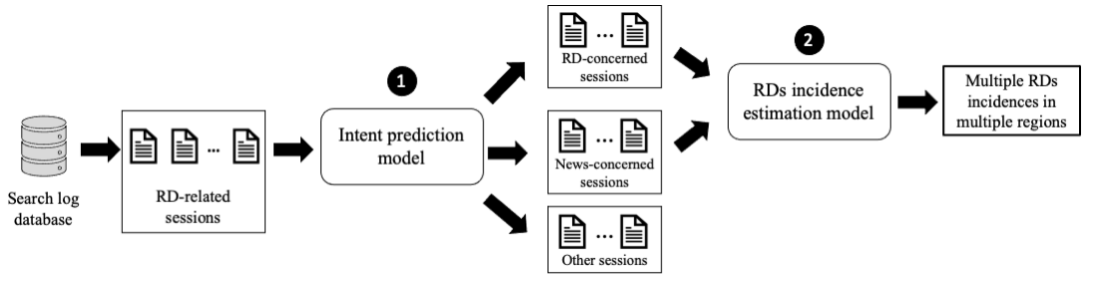
Overview framework of the two-step rare disease (RD) incidences estimation method.

In this study, a two-step method was designed to estimate the incidences of RDs from search engine data. The first step was to distill RD-related search sessions and predict their intents into three categories: RD-concerned, news-concerned, and others. The second step was to estimate multiple RD incidences based on the volume of RD-concerned sessions and news-concerned sessions. Figure 1 shows an overview framework of the proposed two-step method.

The method was applied to search data of 15 RDs in 4 regions in China during 16 seasons from 2016 to 2019. To evaluate the results, we compared the estimated incidences with RD incidences collected from China’s national multicenter clinical database of RDs [5].

Below, we describe the clinical RD incidences data (ie, the ground truth) and search data, followed by descriptions of the first and second steps in more detail, and the experimental settings.

### Data Collection

#### Ethical Approval

This study was approved by the Ethics Committee of Peking Union Medical College Hospital (S-k1790).

#### RD Types and Incidences

All data used in this study were anonymized statistics. A medical professional in the RD scenario helped us select RDs from the Compendium of China’s First List of Rare Diseases (2018) [35]. A total of 15 RDs were selected, containing diseases from diverse departments and had stable long-term data in the registry database. Names and the types of the 15 RDs are listed in Multimedia Appendix 1. More details about the experiments evaluating the influence of RD types are provided in Multimedia Appendix 2.

We obtained the clinical RD incidences data from China’s national multicenter clinical database of RDs [5]. The data set included anonymized confirmed RD cases from 2016 to 2019 reported by more than 300 hospitals across China. The cases were grouped by their diseases (1 of 15 RDs), confirmed time (16 seasons for 2016-2019), and permanent residence locations (one of the four regions in China’s mainland [36]). The RD incidences were determined by dividing the case numbers by the regional population. Ultimately, we obtained incidences of the 15 RDs in 16 seasons (ie, 4 years) in four regions in China.

#### Online Search Data

We collected RD-related queries and their clicked documents from Sogou, one of the top-3 commercial search engines in China. The data were completely anonymized and no personalized information was collected. The side information included the search time and province located by IP address. No specific location was recorded.

First, we collected multisource medical knowledge to form keywords for each RD. Three levels of keywords, ranked by how closely they were associated with the RDs, were considered in our experiments: level 1 included RD-specific keywords, which helped to locate RD-related queries precisely from massive irrelevant queries; level 2 included RD-related nonspecific keywords to indicate how close the queries were related to an RD; and level 3 comprised general medical keywords, which helped determine whether the queries were likely to have medical-related concerns. Experts provided specific keywords about each RD, including disease names, specific genes, and specific treatments, which were defined as level 1 keywords. Based on China’s Guide for the Diagnosis and Treatment of Rare Diseases (2019) [37], we extracted symptoms and pleiotropic treatments for each RD as level 2 keywords. An open medical lexicon [38] on general medical knowledge was treated as level 3 keywords. The lists of level 1 and level 2 keywords are provided in Multimedia Appendix 3, and the level 3 keywords are available from the open lexicon [38].

We matched and saved all queries that contained each level 1 keyword (corresponding to RD names, specific genes, or specific treatments) from all logs of the Sogou search database from 2016 to 2019. Search queries from all level 1 keywords were then merged to constitute the Query Set *Q*, including 2,749,257 queries related to 15 RDs. *Q* could be divided into three categories according to the matched keyword types: 2,615,272 name-related queries, 50,022 gene-related queries, and 83,963 treatment-related queries.

Finally, we introduced the *session* in users’ search process, where a sequence of queries submitted by the same user within 30 minutes formed a session. To be specific, for each query *q* in Query Set *Q* for a user *u*, we backtracked *u*’s query logs before query *q* until the interval between a certain query *q_s_* and the previous query was greater than 30 minutes, and query *q_s_* was then taken as the beginning of the session. We traced *u*’s query logs after *q* until the interval between a certain query *q_e_* and the next query was greater than 30 minutes, and query *q_e_* was then taken as the end of the session. In this way, all sessions with at least one query in *Q* were distilled as the RD-related Session Set *S*, including 1,769,986 sessions. All queries in *S* were then marked with the highest-level keywords they contained. Queries containing level 1 keywords were selected as the *key queries* in the session. In this way, for each query in *S,* we collected the documents that the user clicked under the query. Due to privacy concerns, we only used the URL domains and positions (ie, the rank of the document in the list searched by the query) of the documents.

### Session Intent Prediction

#### Session Intent

Session intent prediction is the first step of our two-step method, which serves to recognize the user intent behind each session in Session Set *S*, providing inputs for the second step. Users’ search intents varied when using the search engine [39]. Although sessions in *S* all mentioned RD-related keywords, they might not come from RD patients or their family members who actually care about RDs. For instance, users might be searching for news, homework assignments, or just out of curiosity. Therefore, it is necessary to distinguish session intents (ie, users’ intents when querying the sessions) in Session Set *S*. We grouped session intents into three categories: *RD-concerned*, *news-concerned*, and *others*. It was considered particularly important to distinguish the news-concerned sessions because breaking news would substantially increase the overall search volume, which would consequently influence the correlation between search volume and disease incidences [22].

#### Feature Extraction

Session-level features and sequences of query-level features were extracted for each session in *S* for predicting the session intent, considering both statistical features and semantic features.

The session-level and query-level statistical features are shown in Table 1. Among them, the Word_freq_change indicated whether a word appeared intensively in queries during a given period. This is a helpful feature to distinguish news-concerned sessions since breaking news will increase the frequency of some uncommon words. The word frequency change *C*(*w_i_*, *t_k_*) of a word *w_i_* in period (ie, season) *t_k_* is defined as:

C(w_i_, t_k_)=[n(w_i_, t_k_)+α]/[∑^K^_j__=1_n(w_i_, t_j_)/K+α]

where *n*(*w_i_*, *t_k_*) is the word frequency of *w_i_* in period *t_j_*, *K* is the number of periods, and *α*=1 for smoothing. At the query level, Word_freq_change is the mean value of all words in the query. At the session level, this feature represents the mean value of all queries in the session.

Both query and document semantic meanings were considered for the semantic features. The frequency of words and document URL domains were calculated separately for each of the three session intent classes. The words and URLs with a high frequency for one intent class and low frequencies for the other two classes were then selected as intent-specific words and URLs. The top 5 intent-specific words and URLs of each intent were selected, forming a set of 15 words and 15 URLs. A 30-dimension session-level vector was then used as a session feature to represent whether each word or URL appeared in a session. Moreover, whether level 1 keywords of each RD appeared in a query was represented with a multihot embedding vector of length 15 (ie, 15 RDs in the data set) as a query feature.

Finally, for a session *S_i_* containing *n_i_* queries, session-level features were concatenated as a vector, 
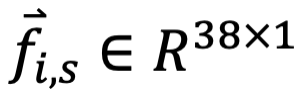
, including 8 dimensions for statistical features and 30 dimensions for semantic features, and query-level features formed a feature sequence 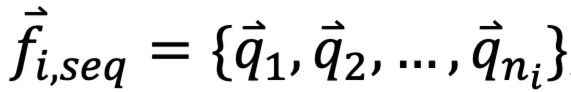, where 
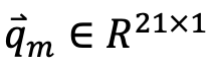
 is the feature vector of the mth query.

**Table 1:** Statistical features used for predicting session intents.

Feature name	Category	Description
Session_len	Session	Session length (ie, number of queries in a session)
Query_type	Query	Level of query (ie, the highest-level keywords a query contains)
Key_num	Session	Number of key (ie, level 1) queries in a session
Q2_num	Session	Number of level 2 queries in a session
Q3_num	Session	Number of level 3 queries in a session
Query_len	Query	Query length (ie, number of words in a query)
Click_num	Query	Number of clicked documents in a query
Sum_click_num	Session	Number of clicked documents in a session
Position_max	Query	Maximum position of clicked documents in the ranking list (set to 0 if no document is clicked)
All_position_max	Session	Maximum of Position_max of all queries in a session
Position_mean	Query	Average position of clicked documents in the ranking list
All_position_mean	Session	Average of Position_mean of all queries with clicked documents in a session
Word_freq_change	Query	Average word frequency change of all words in a query
All_word_freq_change	Session	Average of Word_freq_change of all queries in a session

#### Model Construction

**Figure 2:**
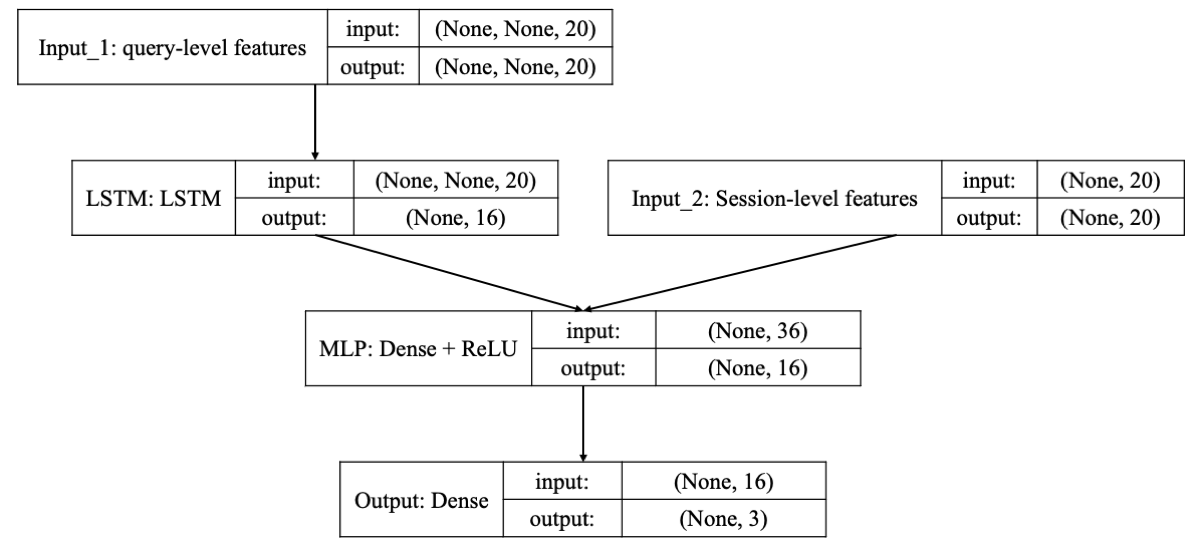
Model structure for session intent prediction. LSTM: long short-term memory; MLP: multilayer perceptron; ReLU: rectified linear unit.

After both sequential features 
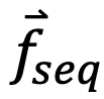
 and vector features 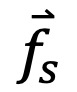 were extracted for intent prediction, a combination of the long short-term memory (LSTM) and multilayer perceptron (MLP) algorithms was used to predict the session intents. The LSTM model is a recurrent neural network that is widely applied for modeling time-series data when the features are sequential [40]. In our work, an LSTM model was employed to transform the sequential features 
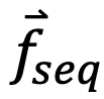
 into a vector 
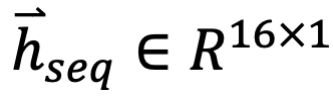
. Subsequently, 
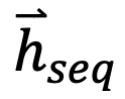
 and 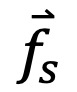 were concatenated and fed into a 1-layer MLP model with a rectified linear unit (ReLU) as an activation function to predict the session intents. The model structure is shown in Figure 2.

### Multiple RD Incidences Estimation

#### Input and Output Construction

To conduct the experiments on incidences estimation for 15 RDs in 16 seasons (ie, 4 years from 2016 to 2019) in 4 regions in China, we constructed the input and output of the second step for multiple RD incidences estimation as shown in Textbox 1.

For the ground truth labels, since the RDs incidence was very low (usually on the 1e–6 order of magnitude), the incidence was rescaled so that the maximum incidence was equal to 1.

**Input and output for multiple rare disease (RD) incidence estimation.**

**Input**

- number of RD-concerned sessions *x_sd_* (*d_i_, l_j_, t_k_*)
- number of news-concerned sessions *x_sn_* (*d_i_, l_j_, t_k_*)

**Output**

- estimated incidence of RD *ŷ*(*d_i_, l_j_, t_k_*), where *d_i_, l_j_*, and *t_k_* indicate the *i*th RD, *j*th region, and *k*th period, respectively

#### LR Model on Multiple RDs and Regions

Following previous research in infoveillance [15,25,34], we chose LR to estimate the incidences of multiple RDs. As the task was to estimate the incidences of multiple RDs in multiple regions, three variants of LR were constructed as incidence estimators.

The first LR model was a general LR, with all of the different RDs and regions estimated with the same set of parameters:

ŷ(d_i_, l_j_, t_k_)=α_d_x_sd_(d_i_, l_i_, t_k_)+α_n_x_sn_(d_i_, l_j_, t_k_)+β,

where *α_d_*, *α_n_*, and *β* are learnable parameters.

The second LR model was an LR with specific parameters for disease (*LR Spec. D.* for short), where estimators of the same RD share parameters:

ŷ(d_i_, l_j_, t_k_)=α_d_(d_i_)x_sd_(d_i_, l_j_, t_k_)+α_n_(d_i_)x_sn_(d_i_, l_j_, t_k_)+β(d_i_)

and *α_n__/d_(d_i_)* and β(d_i_) indicate the learnable parameters for the RD *d_i_*.

The last LR model adopted specific parameters for both disease and regions (*LR Spec. D. L.* for short):

ŷ(d_i_, l_j_, t_k_)=α_d_(d_i_)θ_d_(l_j_)x_sd_(d_i_, l_j_, t_k_)+α_n_(d_i_)θ_n_(l_j_)x_sn_(d_i_, l_j_, t_k_)+β(d_i_)Φ(l_j_)

where *α_n__/d_(d_i_*) and *β(d_i_*) are parameters for disease *d_i_*, and *θ_n__/d_(l_j_*) and *Φ(l_j_*) are parameters for region *l_j_*. All parameters are learnable in training the *LR Spec. D. L.* model.

#### Usefulness of News-Concerned Intents for RD Incidence Estimation

In RDs incidence estimation with session input, news-concerned intents were used as input for the LR models. We aimed to analyze the usefulness of the weights considering news about different diseases (*d_i_*) and regions (*l_j_*) in *LR Spec. D. L.* (ie, *α_sn_*(*d_i_*)*θ_sn_(l_j_)*) by displaying their values and distribution. Moreover, to explore how news-concerned sessions affect RDs incidence estimation dynamically, we tried to find RDs with outbreak news in 2018 and 2019, and display their session numbers, true incidences, and predicted incidences during the study period. In this way, we could illustrate how the news-specific parameters helped reduce the influence of a surge in query volume caused by outbreak news. In the experiment, we selected two diseases: Disease 1 (multiple sclerosis [MS]) and Disease 5 (amyotrophic lateral sclerosis [ALS]). MS represents a class of diseases that has received relatively less attention but gradually attracted public attention, which had related queries on around May 30 every year (ie, International MS Day). ALS is a relatively well-known RD with thousands of RD-concerned and news-concerned sessions, which attracted massive attention when Stephen William Hawking, a world-famous physicist who had ALS, died on March 14, 2018.

### Evaluation Settings

#### Evaluation for Session Intent Prediction (Step 1)

Supervised training was employed to train the session intent prediction model in Figure 2. For the ground truth, a subset *S_anno_* was selected from the session data set *S* to annotate manually. One hundred sessions were randomly selected from each month in 2016 and 2017, forming an *S_anno_* data set of size 2400. Three annotators then labeled the sessions with one of the three intents: RD-concerned, news-concerned, and others. The final intent was voted on by the three annotators. The κ value [41] of the annotations was 0.719, indicating substantial consistency of annotating. Among the 2400 annotated sessions, 502 were RD-concerned, 143 were news-concerned, and 1755 belonged to the others category. Thus, a considerable percentage of sessions were not RD-concerned, indicating that it is necessary to distinguish the session intents. The 2400 sessions were randomly divided into a training set, validation set, and test set at an 8:1:1 ratio.

For model implementation, Python 3.6.13 was used for modeling and evaluation. Pytorch 1.7.1 was used as the framework for training the models. Macro-F1, accuracy, and F1 scores for each intent were used for performance evaluation.

#### Evaluation of Multiple RDs Incidence Estimation (Step 2)

For comparison, we also constructed query data as the input for RDs incidence estimation. The query input comprised the numbers of name-related, gene-related, and treatment-related queries of different RDs, regions, and periods. The structures of LR variants for the query input are the same as the equations presented in the previous subsection.

We compared different input types and LR models on the data set from 2016 to 2019, where data in 2016 and 2017 constituted the training set, data in 2018 served as the validation set, and data in 2019 served as the test set. The root mean square error (RMSE) and relative error rate (RER) were utilized for performance evaluation to obtain both the absolute error and relative error of the models:



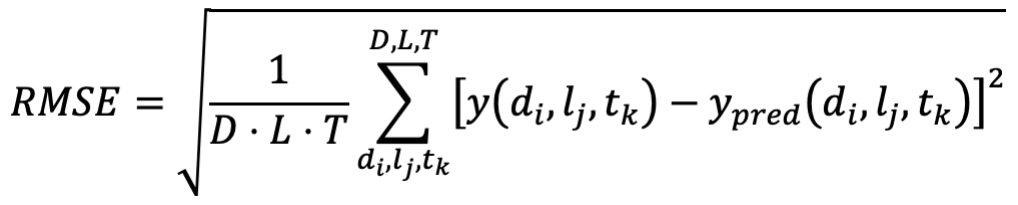





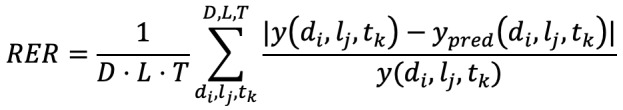



where *y_pred_*(*d_i_*, *l_j_*, *t_k_*) is the predicted output of LR models.

All experiments were conducted in the Python 3.6.13 environment and all methods were implemented with the Pytorch 1.7.1 library. Models were trained with the Adam optimizer until convergence on the validation set with a maximum of 1000 epochs.

## Results

### Summary Statistics of RDs Incidence and RD-Related Search Data

In general, the RDs incidence data set included more than 80,000 incidences from 2016 to 2019 in China (due to data privacy concerns, the specific number of incidences is not reported). The RD-related search data set included 2,749,257 RD-related queries and 1,769,986 sessions from 1,380,186 users. It is worth noting that repeated search was not a serious problem in our data set. On average, each user had 1.282 sessions, most users (n=1,193,362, 86.46%) had only one session, and 97.75% (n=1,349,105) of users contributed less than four sessions. This is mainly due to two reasons. First, the sessions grouped RD-related search queries that were submitted by a user over a short period of time; therefore, repeated sessions were less common for RD patients in our data set. Second, we distilled RD-related sessions by specific keywords for RDs (ie, level 1 keywords), and the provided results might be sufficiently clear that there was no need to repeat the search. Therefore, we adopted the intent prediction and incidence estimation tasks at the session level rather than the user level.

Furthermore, we considered four regions in our data set, which divided 31 provinces in China’s mainland into four parts: East, West, Central, and Northeast. The populations of the four regions were 535.6 million, 378.1 million, 369.9 million, and 108.5 million, with gross domestic products of 7109 billion dollar, 2752 billion dollar, 2899 billion dollar, and 797 billion dollar, respectively (average of 4 years). In the RDs incidence data set, the sum of the incidences of 15 RDs was the highest in the West, followed by the East, Central, and Northeast regions. The incidence of different RDs varied among the four regions. For instance, MS and hemophilia had the largest incidences in the West, whereas ALS was the most frequently registered disease in the East. In the RD-related search data set, the average session and query numbers of the 4 years were 225,906.5 and 1,023,152.0 for the East; 91,357.5 and 413,361.3 for the West; 94,151.8 and 429,708.5 for the Central region; and 31,080.8 and 141,278.0 for the Northeast, respectively.

Generally, the East had the largest population, the most developed economy, and, accordingly, the highest number of queries and sessions. Overall, the session volume was proportional to the population. However, regional reported RD incidences and population did not always match, since the incidence of an RD in a given region might relate to whether it is a family genetic disease in the region, the diagnosis technique of the disease in that region, and other factors. Therefore, we considered the effect of region variables on the RD incidence estimation specifically.

### Performance of Session Intent Prediction

The first-step session intent prediction was evaluated with the human-annotated test set of 240 sessions. In the three-category classification task, the model had a macro-F1 value of 0.452 and an accuracy of 0.682 on the test set. The F1 scores for RD-concerned sessions, news-concerned sessions, and other sessions were 0.397, 0.353, and 0.606, respectively. Some representative sessions with different intents are shown in Multimedia Appendix 4. All of the sessions were correctly predicted with the intent prediction model.

Finally, the model was applied to predict the intents of all 1,769,986 sessions in Session Set *S*, resulting in 426,031 RD-concerned sessions, 115,016 news-concerned sessions, and 1,228,939 other sessions. The RD-concerned and news-concerned sessions were grouped by their RDs, regions, and periods to form the session inputs *x_sd_*(*d_i_*, *l_j_*, *t_k_*) and *x_sn_*(*d_i_*, *l_j_*, *t_k_*) for comparing and estimating RDs incidence.

### Performance of Incidence Estimation

#### Overall Performance

The incidence estimation results of different input types and LR models are shown in Table 2. Each experiment was repeated five times with different random seeds, and the average result and 95% CIs are reported. The null hypothesis was that there was no difference between the estimation results using query and session as the input. A two-sided *t*-test was performed on the results with different input types on the same model, and the *P* values are also reported in Table 2.

Session input had significantly better performance than query input on all models and metrics, which indicated the usefulness of considering search session intents in the RDs incidence estimation task. Comparing different models, *LR Spec. D. L.* exhibited the best performance, with RER=0.365 on session input and RER=0.511 on query input. However, the 95% CI was large. The instability was mainly due to the relatively large number of parameters in *LR Spec. D. L.* Further detailed comparison between session input and query input are shown in Multimedia Appendix 5.

**Table 2:** Relative error rate (RER) and root mean square error (RMSE) of rare disease incidence prediction with different linear regression (LR) models and input types.

Model		RER		RMSE	
		Average value (95% CI)	*P* value	Average value (95% CI)	*P* value
**General LR**					
	Query input	0.998 (0.997-0.999)	<.001	0.042 (0.042-0.042)	<.001
	Session input	0.864 (0.848-0.879)		0.039 (0.03-0.039)	
**LR Spec. D.^a^**					
	Query input	0.887 (0.872-0.903)	<.001	0.037 (0.037-0.038)	<.001
	Session input	0.720 (0.676-0.764)		0.030 (0.028-0.032)	
**LR Spec. D. L.^b^**					
	Query input	0.511 (0.377-0.645)	.01	0.023 (0.017-0.029)	.008
	Session input	0.365 (0.341-0.388)		0.017 (0.016-0.017)	

^a^Spec. D.: specific disease.

^b^Spec. D. L.: specific disease and location.

#### Usefulness of News-Concerned Intents for RDs Incidence Estimation

**Figure 3:**
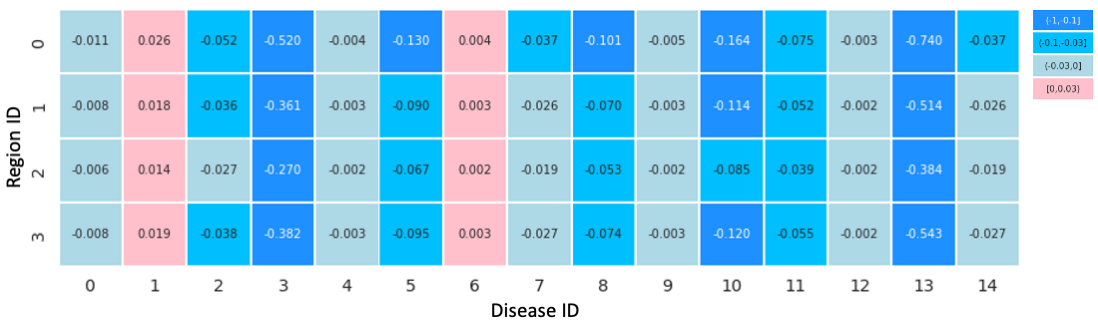
Weights of news-concerned session numbers in estimating the rare diseases incidence with the linear regression specific disease and location (LR Spec. D. L.) model.

The weights considering news about different diseases *d_i_* and regions *l_j_* in the *LR Spec. D. L.* model (ie, *α_sn_*(*d_i_*)*θ_sn_*(*l_j_*)) are shown in Figure 3. The weights of news-concerned sessions were primarily negative, which confirmed our hypothesis that the effect of news should be deducted from the disease estimation, consistent with the findings of Xu et al [22]. The two outliers were Diseases 1 and 6, which had very small but positive parameters. There were too few news-concerned sessions (a few dozen) for these two diseases, and therefore they had little impact on the results. Moreover, since the volumes of search sessions and incidence were distinct, the magnitude of parameters varied among RDs.

**Figure 4:**
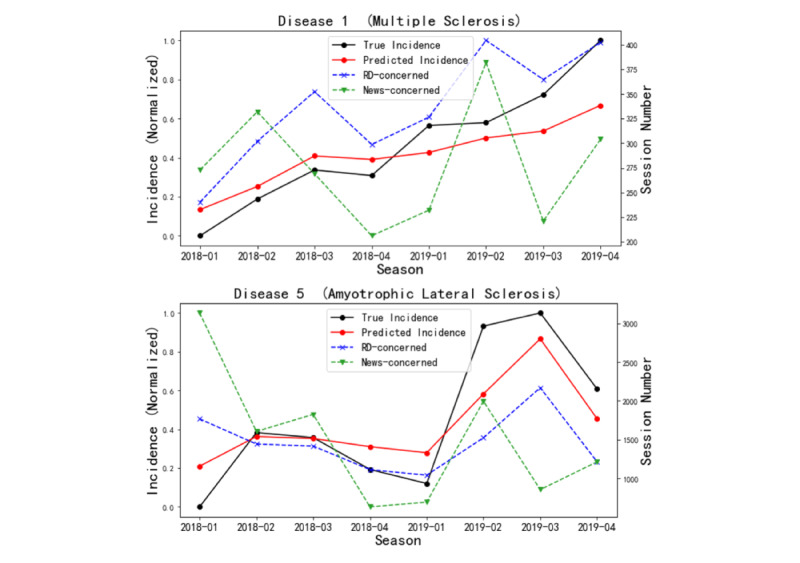
News-concerned session numbers, rare disease (RD)-concerned session numbers, and RDs true incidence and predicted incidence (normalized to the range of 0 to 1) of each season during 2018 and 2019 for Disease 1 (multiple sclerosis) and Disease 5 (amyotrophic lateral sclerosis).

To explore how news-concerned sessions affect RDs incidence estimation dynamically, we display two cases of RDs for Disease 1 (MS) and Disease 5 (ALS) in Figure 4. News-concerned session numbers, RD-concerned session numbers, and the true and predicted incidence (normalized to the range of 0 to 1) of RDs for each season during 2018 and 2019 are shown. Figure 4 demonstrates that outbreak news could be predicted with the intent prediction model, and the predicted incidence was corrected from the high query volume when the news-concerned sessions were considered. For MS, two peaks in news-concerned session numbers arose in the second seasons of 2018 and 2019 around May 30, International MS Day. By contrast, since the MS incidence was certainly not affected by MS Day, considering news-concerned sessions would reduce noise in session numbers for incidence estimation. News-concerned ALS sessions showed a noticeable peak in the 1st season in 2018, after Stephen William Hawking died on March 14, 2018. After considering the number of news-concerned sessions, the result was less affected by the outbreak news.

## Discussion

### Principal Findings

The RD incidence estimation experiment on 15 RDs in 4 regions of China showed that RDs could be estimated with search engine logs, especially search session data. The RER of RDs incidence estimation was 0.365 for the session input and 0.511 for the query input. Considering the sparsity of RD cases, the RDs incidence estimation performance is encouraging.

The first step predicted session intents with a deep neural model. The prediction results indicated the necessity to distinguish the user intents in searching sessions. Among 1,769,986 RD-related sessions, only 426,031 (24.07%) were RD-concerned and 1,228,939 (69.43%) belonged to other intents. By identifying sessions concerned with RDs, irrelevant queries were effectively filtered from the data.

The second step, multiple RDs incidence estimation with LR, demonstrated that considering the volume of sessions rather than RD-related queries was significantly more helpful for disease estimation in most RDs and regions, as shown in Table 2 and Multimedia Appendix 5. Compared with queries, session intents helped estimate RDs incidence with an error decrease of 28.57% in terms of RER (*P*=.01). This illustrates the significant contribution of considering search sessions with more context for RD incidence detection. Moreover, as shown in Figure 3 and Figure 4, considering news-concerned session numbers in RDs incidence estimation was necessary and helpful. When we considered the types of RDs (Multimedia Appendix 2), no significant differences were revealed between the similarity within each RD type and the similarity between different types. Adding RD type–specific parameters to the incidence estimation model also did not improve performance. Since the incidence and search query for RDs were both too sparse, their distributions might be less correlated with RD types. Moreover, RDs are often associated with genetics, and genetic variants vary among RDs of the same types, resulting in different distributions. The role of RD types is therefore considered to be relatively less important in RD-related infoveillance.

### Comparison With Prior Work

To our knowledge, this study is the first to apply infoveillance in RDs incidence estimation, which provides a novel method to understand RDs. Compared with prior research on utilizing search engine data to estimate other diseases, a novel aspect of this study is that we considered the session context about disease-related queries and then utilized session intents to replace query volume for disease incidence estimation. Session inputs showed significant improvement on the RDs incidence estimation task. Although the sparsity of RD-related queries inspired the use of session information, the two-step method can be effectively transferred to other search engine–based disease detection and estimation tasks, as data noise pervasively exists online.

### Limitations

This study has several limitations. First, the current data from the national multicenter clinical database of RDs were collected by retrospective reports. Due to the difficulty of RD diagnosis and the limited support of International Classification of Diseases 10th Revision codes for RDs, there might be delayed or unreported cases in the database. Therefore, the overestimations of incidence might reflect unreported cases, which was neglected in our analysis and discussions. In the future, it would be helpful to revisit patients in overestimated RDs and regions with privacy protection.

Second, 15 RDs with stable long-term data in the registry database were utilized for our experiments. These experiments could be applied to other RDs, whereas some RDs might not be estimated with our proposed methods, such as those with unclear symptoms, too low incidence, and low public awareness. Extending this method to more RDs and finding the boundary is promising future work.

Third, the level 1 keywords used for matching RD-related queries were provided by medical experts, which was time-consuming and might reflect knowledge bias. In the future, we will test automatic keyword discovery methods for RD-related keyword discovery.

Finally, a simple combination of LSTM and MLP was adopted for intent prediction in this study as the first attempt to integrate session intents in RDs incidence estimation. Since the numbers of RD-concerned and news-concerned sessions were much smaller than the numbers of sessions about other intents, the F1 scores of intent prediction about RD-concerned and news-concerned sessions were limited (0.397 and 0.353, respectively). Although challenging, accurate intent prediction is essential for capturing RD-concerned sessions precisely. Therefore, we aim to design neural predictors with more sophisticated network structures and more features about the sessions and queries to improve the session intent prediction accuracy, especially for RD-concerned and news-concerned sessions.

### Conclusions

In this study, an experiment on multiple RDs in multiple regions showed that it is possible to estimate RDs incidence with online search engine data. The two-step estimation method illustrates promising performance improvement when session intents are considered in the RDs incidence estimation task. The use of session information can be transferred to infoveillance on other diseases.

This study did not aim to replace the clinical RD registry systems with search engine–based estimation. The two-step RDs incidence estimation model was designed as a supplement and prewarning method. For instance, if the model overestimates an RD in a region, this can remind experts of possible missing records from clinical registries or lack of medical support in the region. This method could help provide information for allocating medical resources and RD-related policy-making in the future. Moreover, with privacy protection, the method could offer advice to RD-concerned users of appropriate medical aids such as hospitals or institutes specialized in certain RDs. In conclusion, this study provides a promising method for understanding and locating RDs.

## Appendix

Multimedia Appendix 1 Rare disease (RD) names and types.

Multimedia Appendix 2 Influence of disease types on rare diseases incidence estimation.

Multimedia Appendix 3 Keyword lists.

Multimedia Appendix 4 Representative sessions with different intents.

Multimedia Appendix 5 Comparison between session input and query input for rare disease incidence estimation.

## Abbreviations

ALS: amyotrophic lateral sclerosis
LR: linear regression
LSTM: long short-term memory
MLP: multilayer perceptron
MS: multiple sclerosis
RD: rare disease
ReLU: rectified linear unit
RER: relative error rate
RMSE: root mean square error

## Data Availability

The data sets generated and/or analyzed during the current study are not publicly available due to patients’ privacy concerns, but are available from the corresponding author on reasonable request.
