# Supplementary material for: Estimating Rare Disease Incidences With Large-scale Internet Search Data: Development and Evaluation of a Two-step Machine Learning Method
Source: JMIR Infodemiology. 2023 Apr 28;3:e42721. doi: 10.2196/42721 (PMC10182453; doi:10.2196/42721)
Supplement: Multimedia Appendix 1 [file infodemiology_v3i1e42721_app1.docx]

## Multimedia Appendix 1: RD Names and Types

IDs, names, and types of 15 rare diseases used in our experiments.

| **Disease Id** | **Disease Name** | **Disease Type** |
| --- | --- | --- |
| 0 | Multiple Sclerosis | neuromuscular disease |
| 1 | Multiple System Atrophy | neuromuscular disease |
| 2 | Idiopathic Pulmonary Arterial Hypertension | cardiopulmonary disease |
| 3 | Idiopathic Pulmonary Fibrosis | cardiopulmonary disease |
| 4 | Systemic Sclerosis | skin disease |
| 5 | Amyotrophic Lateral Sclerosis | neuromuscular disease |
| 6 | Hepatolenticular Degeneration (Wilson Disease) | hematological and metabolic disease |
| 7 | Autoimmune Encephalitis | neuromuscular disease |
| 8 | Hemophilia | hematological and metabolic disease |
| 9 | Neuromyelitis Optica | neuromuscular disease |
| 10 | Retinoblastoma | opthalmological disease |
| 11 | Retinitis Pigmentosa | opthalmological disease |
| 12 | Progressive Muscular Dystrophy | neuromuscular disease |
| 13 | Hereditary Epidermolysis Bullosa | skin disease |
| 14 | Paroxysmal Nocturnal Hemoglobinuria | hematological and metabolic disease |
