# Supplementary material for: Estimating Rare Disease Incidences With Large-scale Internet Search Data: Development and Evaluation of a Two-step Machine Learning Method
Source: JMIR Infodemiology. 2023 Apr 28;3:e42721. doi: 10.2196/42721 (PMC10182453; doi:10.2196/42721)
Supplement: Multimedia Appendix 2 [file infodemiology_v3i1e42721_app2.docx]

## Multimedia Appendix 2: Influence of Disease Types on RDs incidence estimation

### Methods

As RDs belong to various clinical departments, different types of RDs were included in the dataset. In this subsection, we tried to explore whether RDs with the same type would share similar characteristics and whether the types of RDs would help estimate RDs incidence. According to expert annotations, the 15 RDs considered in our experiments could be grouped into five types, as detailed in Multimedia Appendix 1.

Firstly, we sought to explore whether RDs of the same type were more similar to each other than to different types of RDs. ANOSIM (Analysis of similarities) was adopted to test it. We conducted ANOSIM on the similarities of three kinds of data used for incidence estimation, i.e., RD-concerned session numbers , News-concerned session numbers , and RD incidence . The similarity between two RDs was defined as the correlation of their session numbers or RD incidence among different regions and periods.

Then, we tried to differentiate the types of RDs in the incidence estimation model. We proposed a variant of *LR Spec. D. L.*, named *LR Spec. D.L.Dt.*, to consider RD type-specific parameters. Since differences were found in RD-concerned session numbers and RD incidence by ANOSIM, RD type-specific learnable parameters were applied to the coefficient of and the intercept of regression, respectively,

Where indicates the disease type of the *i*th disease, is the RD type-specific coefficient, and is the RD type-specific intercept.

The same training strategy and evaluation metrics as LR Spec. D. L. were adopted.

### Results

Firstly, we adopted ANOSIM to explore whether RDs of the same type were more similar to each other than to different types of RDs. The ANOSIM test statistic R was 0.182 for RD-concerned , 0.085 for News-concerned , and 0.203 for RD incidence , respectively, indicating differences with an overlap of RD-concerned session numbers and RD incidences, and almost no differences of News-concerned session numbers. Moreover, the results were not significant on all three tests, indicating that similarity within the RD types was not significantly greater than the similarity between RD types.

Then, we tried to differentiate the types of RDs in the incidence estimation model with *LR Spec. D.L.Dt.*, which considered RD type-specific parameters when estimating the incidence. Average results of five repeated experiments showed that RER of *LR Spec. D. L. Dt.* was 0.418 with CI=[0.372, 0.501], and the RMSE was 0.021 with CI=[0.019, 0.025]. The performance of *LR Spec. D.L.Dt.* is no better than LR *Spec. D.L.*

Therefore, from both analysis and modeling results, we found the contribution of disease types was limited for RD incidence estimation with RD-related search data.
