# Supplementary material for: Estimating Rare Disease Incidences With Large-scale Internet Search Data: Development and Evaluation of a Two-step Machine Learning Method
Source: JMIR Infodemiology. 2023 Apr 28;3:e42721. doi: 10.2196/42721 (PMC10182453; doi:10.2196/42721)
Supplement: Multimedia Appendix 3 [file infodemiology_v3i1e42721_app3.docx]

## Multimedia Appendix 3: Keyword Lists

Ids and names of the 15 RDs that we selected are as follows:

Three levels of keywords were utilized to match the queries in our experiments, including 285 *Level 1* keywords about RD-specific terms, 1886 *Level 2* keywords about RD-related non-specific terms, and 13531 *Level 3* general medical keywords from an open lexicon. The lists of Level 1 and Level 2 keywords are shown as follows. Since we conducted the experiments on the Chinese corpus, all keywords are in Chinese.

Level 1 keywords for each of 15 rare diseases.

| **Disease Name** | **Level 1 keywords** |
| --- | --- |
| Multiple Sclerosis | 多发性硬化,多发硬化,multiple sclerosis,特立氟胺,奥巴捷,干扰素β,干扰素beta,干扰素 beta,干扰素贝塔,利比,倍泰龙,MS |
| Multiple System Atrophy | 多系统萎缩,橄榄体脑桥小脑萎缩,橄榄脑桥小脑萎缩,橄榄体脑桥小脑萎,橄榄脑桥小脑萎,纹状体黑质变性,纹状黑质变性,Shy-Drager综合征,Shy-Drager综合症,Shy Drager综合征,Shy Drager综合症,ShyDrager综合征,ShyDrager综合症,Shy-Drager 综合征,Shy-Drager 综合症,Shy Drager 综合征,Shy Drager 综合症,ShyDrager 综合征,ShyDrager 综合症,Shy-Drager病,Shy Drager病,ShyDrager病,Shy-Drager 病,Shy Drager 病,ShyDrager 病,multiple system atrophy,olivopontocerebellar atrophy,striatonigral degeneration,Shy-Drager syndrome,Shy Drager syndrome,ShyDrager syndrome,MSA |
| Idiopathic Pulmonary Arterial Hypertension | 特发性肺动脉高压,特发肺动脉高压,特发性肺高压,特发肺高压,原发性肺动脉高压,原发肺动脉高压,原发性肺高压,原发肺高压,idiopathic pulmonary artery hypertension,idiopathic pulmonary hypertension,primary pulmonary artery hypertension,primary pulmonary hypertension,IPAH |
| Idiopathic Pulmonary Fibrosis | 特发性肺纤维化,特发肺纤维化,特发性肺纤维,特发肺纤维,特发性肺纤化,特发肺纤化,寻常型间质肺炎,寻常间质肺炎,隐源性致纤维化性肺泡炎,隐源性致纤维化肺泡炎,隐源致纤维化肺泡炎,隐源致纤维化性肺泡炎,隐源性致纤维性肺泡炎,隐源性致纤维肺泡炎,隐源致纤维肺泡炎,隐源致纤维性肺泡炎,隐源性致纤化性肺泡炎,隐源性致纤化肺泡炎,隐源致纤化肺泡炎,隐源致纤化性肺泡炎,idiopathic pulmonary fibrosis,usual interstitial pneumonia,cryptogenic fibrosing alveolitis,尼达尼布,吡非尼酮,IPF |
| Systemic Sclerosis | 系统性硬化症,系统性硬化征,系统硬化症,系统硬化征,RNA聚合酶Ⅲ抗体阳性,RNA聚合酶3抗体阳性,RNA聚合酶三抗体阳性,RNA 聚合酶Ⅲ抗体阳性,RNA 聚合酶3抗体阳性,RNA 聚合酶三抗体阳性,RNA聚合酶Ⅲ抗体阳,RNA聚合酶3抗体阳,RNA聚合酶三抗体阳,RNA 聚合酶Ⅲ抗体阳,RNA 聚合酶3抗体阳,RNA 聚合酶三抗体阳,拓扑异构酶Ⅰ抗体阳性,拓扑异构酶1抗体阳性,拓扑异构酶一抗体阳性,拓扑异构酶Ⅰ抗体阳,拓扑异构酶1抗体阳,拓扑异构酶一抗体阳,着丝点抗体阳性,着丝点抗体阳,systemic sclerosis,Scl-70,Scl70,Scl 70,SSc |
| Amyotrophic Lateral Sclerosis | 肌萎缩侧索硬化,肌萎缩侧索硬,肌萎缩厕所硬化,肌萎缩厕所硬,肌肉萎缩侧索硬化,肌肉萎缩侧索硬,肌肉萎缩厕所硬化,肌肉肉萎缩厕所硬,肌肉萎缩性侧索硬化,肌肉萎缩性侧索硬,肌肉萎缩性厕所硬化,肌肉萎缩性厕所硬,肌萎缩性侧索硬化,肌萎缩性侧索硬,肌萎缩性厕所硬化,肌萎缩性厕所硬,运动神经元病,渐冻症,渐冻征,卢伽雷病,lou gehrig病,lou gehrig 病,amyotrophic lateral sclerosis,motor neuron disease,lou Gehrig’s disease,lou Gehrig disease,lou Gehrig's disease,lou Gehrig s disease,lou Gehrigs disease,力如太,利鲁唑,ALS,SOD1,TDP-43,TDP43,TDP 43,FUS,C9ORF72,TARDBP |
| Hepatolenticular Degeneration(Wilson Disease) | 肝豆状核变性,肝豆核变性,威尔逊氏病,威尔逊病,Wilson病,Wilson 病,hepatolenticular degeneration,wilson disease,K-F环,K-F 环,KF环,KF 环,K F环,K F 环,铜蓝蛋白低,ATP7B |
| Autoimmune Encephalitis | 自身免疫性脑炎,自身免疫脑炎,autoimmune encephalitis,NMDAR,IgLON5,DPPX,AMPAR,GFAP,LGI1,GABABR |
| Hemophilia | 血友病,hemophilia,重组人凝血因子Ⅷ,重组人凝血因子8,重组人凝血因子八,任捷,拜科奇 |
| Neuromyelitis Optica | 视神经脊髓炎,视神经脊髓炎谱系疾病,devic病,devic 病,德维克病,neuromyelitis optica spectrum disorders,neuromyelitis optica,水通道蛋白4抗体阳性,水通道蛋白4抗体阳,水通道蛋白四抗体阳性,水通道蛋白四抗体阳,水通道蛋白抗体Ⅳ阳性,水通道蛋白Ⅳ抗体阳,NMOSD,NMO,AQP4 |
| Retinoblastoma | 视网膜母细胞瘤,retinoblastoma,RB-1,RB1,RB 1 |
| Retinitis Pigmentosa | 视网膜色素变性,视杆-视椎细胞营养不良,视杆视椎细胞营养不良,视杆 视椎细胞营养不良,毯层视网膜变性,色素性视网膜病,色素视网膜病,retinitis pigmentosa,rod-cone dystrophy,tapetoretinal degeneration,pigmentary retinopathy,RP,CYP4V2,RHO,USH 2A,RPGR,EYS |
| Progressive Muscular Dystrophy | 进行性肌营养不良,进行肌营养不良,progressive muscular dystrophy,Duchenne型肌营养不良,Duchenne肌营养不良,Duchenne 型肌营养不良,Duchenne 肌营养不良,Becker型肌营养不良,Becker肌营养不良,Becker 型肌营养不良,Becker 肌营养不良,面肩肱型肌营养不良,面肩肱肌营养不良,先天性肌营养不良,先天肌营养不良,肌营养不良征,肌营养不良症,DMD,SMCHD1 |
| Hereditary Epidermolysis Bullosa | 遗传大疱性表皮松解症,遗传大疱表皮松解症,大疱表皮松解症,半桥粒大疱表皮松解症,hereditary epidermolysis bullosa,遗传性大疱性表皮松解征,遗传性大疱表皮松解征,大疱性表皮松解征,半桥粒大疱性表皮松解征,遗传性大疱性表皮松解症,遗传性大疱表皮松解症,大疱性表皮松解症,半桥粒大疱性表皮松解症,KRT5,KRT14,PLEC1,DSP,COL7A1,LAMA3,LAMB3,LAMC2,COL17A1,ITGB4,ITGA6 |
| Paroxysmal Nocturnal Hemoglobinuria | 阵发性睡眠性血红蛋白尿,阵发睡眠性血红蛋白尿,阵发性睡眠血红蛋白尿,阵发睡眠血红蛋白尿,阵发睡眠性血红蛋白尿症,阵发睡眠血红蛋白尿症,阵发夜间血红蛋白尿症,paroxysmal nocturnal hemoglobinuria,阵发性夜间血红蛋白尿,阵发夜间血红蛋白尿,阵发性睡眠性血红蛋白尿征,阵发性睡眠血红蛋白尿征,阵发性夜间血红蛋白尿征,阵发性睡眠性血红蛋白尿症,阵发性睡眠血红蛋白尿症,阵发性夜间血红蛋白尿症,PNH,PIG-A,PIGA,PIG A |

Level 2 keywords for each of 15 rare diseases.

| **Disease Name** | **Level 1 keywords** |
| --- | --- |
| Multiple Sclerosis | 中枢神经系统,炎性脱髓鞘,脑室,近皮质,视神经,脊髓,脑干,小脑,空间多发,时间多发,免疫反应发病,多急性起病,视力下降,复视,肢体感觉障碍,肢体运动障碍,共济失调,膀胱功能障碍,直肠功能障碍,复发缓解型,亚急性起病,继发进展型,原发进展型 ,进展复发型,实验室检查,脑脊液,寡克隆区,白细胞,CSF-IgG,IgG鞘内合成,琼脂糖,电聚焦,免疫印迹技术,lyme病,Lyme 病,神经梅毒,亚急性硬化性全脑炎,人类缺陷病毒感染,结缔组织病,MRI,脑室周围,胼胝体,半卵圆中心,深部白质,基底节,胼胝体排列,矢状位图像,Dawson 手指征,脊髓病灶,颈髓,脊髓肿胀,T2 加权序列,Dawson手指征,McDonald诊断标准,McDonald 诊断标准,急性播散性脑脊髓炎,临床孤立综合征,侧脑室周围,皮层或近皮层,颅内病变,视神经脊髓炎,CADASIL,散性脑脊髓炎,假瘤型脱髓鞘病,血管病,多发性腔隙性脑梗死,烟雾病,血管畸形,莱姆病,梅毒,脑囊虫,热带痉挛性截瘫,艾滋病,进行性多灶性白质脑病,系统性免疫病,系统性红斑狼疮,白塞综合征,系统性血管炎,原发性中枢神经系统血管炎,遗传代谢性疾病,脑白质营养不良,线粒体脑肌病,维生素 B12 缺乏,叶酸缺乏,特异性治疗,急性期治疗,缓解期治疗,疾病修正治疗,神经缺损,运动障碍,视力下降,甲泼尼龙冲击治疗,静脉滴注,口服泼尼松,二线治疗,MRI 病变,免疫球蛋白治疗,IVIG治疗,不良反应,电解质紊乱,上消化道出血,骨质疏松,股骨头坏死,血浆置换,清除自身抗体,调节免疫,急性重症,激素治疗,静注人免疫球蛋白,糖皮质激素,干扰素β,聚乙二醇干扰素,醋酸格列默,芬戈莫德,富马酸二甲酯,单克隆抗体,阿仑单抗,奥瑞珠单抗,非特异性免疫抑制剂,米托蒽醌,综合治疗,功能康复锻炼,维生素D,雌激素,那他珠单抗,阿伦单抗,慢性疼痛,痛性痉挛,膀胱直肠功能障碍,认知障碍,病友,关爱中心,互助家园,MS |
| Multiple System Atrophy | 神经退行性疾病,帕金森病,自主神经功能衰竭,帕金森症,小脑性共济失调,锥体束征,脑病理,少突胶质细胞胞浆,嗜酸性包涵体,突触核蛋白,橄榄萎缩,脑桥萎缩,小脑萎缩,黑质纹状体变性,外源性毒物接触史,有机溶剂,塑料单体,锥体束损害,自主神经系统损害,自主神经损害,心血管功能障碍,直立性低血压,排尿障碍,尿失禁,合并性功能障碍,快速眼动期,睡眠障碍,睡眠行为障碍,MSA-P型,MSA-C型,神经功能障碍,排汗系统障碍,壳核裂隙,氟-脱氧葡萄糖,F-FDG PET,自主神经检查,动态血压监测,直立倾斜试验,呼吸电子喉镜,声带麻痹,多导睡眠监测,尿动力,泌尿系超声,吞咽造影,结肠通过时间,肛门括约肌肌电图,体温调节,泌汗试验,定量泌汗轴索反应,帕金森型,小脑型,口面肌张力障碍,颈部前屈,严重躯干前曲,Pisa 综合征,pisa 综合征,躯干肌张力障碍,手或足挛缩,吸气性叹息,发音困难,构音障碍,咬字不清,说话含糊,鼻音过重,节律异常,新发打鼾,加重打鼾,打鼾加重,静止性震颤,周围神经病变,共济失调,帕金森综合征,痴呆,白质损害,多发性硬化,非药源性幻觉,手足冰冷,强笑强哭,动作性震颤,肌阵挛样,姿势性震颤,脑结构损害,ILOCA,自主神经功能障碍,MSA-P 亚型,眼肌麻痹,皮质基底节变性,左旋多巴,自主神经障碍,核上性眼球活动障碍,假性球麻痹,中轴躯干性肌强直,异己手（肢）综合征,异己手综合征,异己肢综合征,认知功能障碍,皮质感觉障碍,不对称性肌强直,刺激敏感,肌阵挛,路易体痴呆,进行性核上性麻痹,抗帕金森病药物,升压药,复方左旋多巴,金刚烷胺,神经源性膀胱,急迫性尿失禁,抗胆碱能药物,认知功能下降,肉毒素治疗,尿潴留,间歇性自家导尿,膀胱造瘘,胆碱能药物或α受体拮抗剂,非药物治疗,药物治疗,米多君,屈昔多巴,氟氢可的松,短效降压药,夜间吸气性喉鸣,肉毒素注射,氯硝西泮,渗透性膨胀通便剂,控制排尿药,可用持续正压通气,尿排空障碍,黑质纹状体,多巴胺能纤维脱失,喘鸣,巴宾斯基征,腱反射活跃,病友,关爱中心,互助家园,MSA |
| Idiopathic Pulmonary Arterial Hypertension | 肺血管阻力,恶性肺血管疾病,右心功能衰竭,BMPR2基因,BMP9基因,KCNK3基因,ACVRL1基因,ENG基因,SMAD9基因,BMPR1B基因,TBX4基因,阿米雷司,新发IPAH,活动后气短,头晕,乏力,胸痛,胸闷,心悸,黑矇晕厥,眩晕,心搏量下降,现肺动脉瓣第二心音亢进,三尖瓣关闭不全,三尖瓣区收缩期杂音,颈静脉充盈,颈静脉怒张,肝脏肿大,下肢水肿,多浆膜腔积液,黄疸,发绀,心内分流性畸形,胸部ct,ct肺动脉造影,肺间质病变,肺血管畸形,肺动静脉瘘,肺动脉瘤,肺动脉夹层,肺动/静脉阻塞,肺动脉阻塞,肺静脉阻塞,急性肺栓塞,CTEPH,大动脉炎,肺动脉肿瘤,纤维纵隔炎,超声心动图,肺动脉收缩压,右心大小,心内分流,左心疾病,肺实质疾病,气道疾病,外周小气道功能障碍,弥散功能,肺通气/灌注显像,肺通气灌注显像,肺通气显像,肺灌注显像,慢性血栓栓塞,右心导管,漂浮导管,急性血管扩张,肺动脉造影,肺动脉狭窄,肺静脉狭窄,肺血管疾病,门脉高压,结缔组织病,血流动力学诊断,肺动脉平均压,肺小动脉楔压,活动耐量减低,外周水肿,左心衰,心包疾病,腔静脉病变,肝脏疾病,先天性分流性心脏病,感染性疾病,血液性疾病,抗凝治疗,靶向药物,液体潴留,利尿剂,外周血氧饱和度,氧疗,心排血量降低,房颤,地高辛,钙通道阻滞剂,急性肺血管扩张,地尔硫卓,硝苯地平,氨氯地平,内皮素,波生坦,安立生坦,马昔腾坦,5型磷酸二酯酶,环磷酸鸟苷,肺血管舒张,西地那非,他达拉非,伐地那非,前列环素,内源性血小板聚集,依前列醇,伊洛前列素,曲前列尼尔,贝前列素,鸟苷酸环化酶,利奥西呱,司来帕格,有序贯联合治疗,起始联合治疗,球囊扩张房间隔造口术,心输血量增加,改善体循环氧气运输,降低交感神经过度兴奋,肺移植,心肺联合移植,血流动力学受损,运动耐量,囊性纤维化,血吸虫病,心血管内科,病友,关爱中心,互助家园,IPAH |
| Idiopathic Pulmonary Fibrosis | 肺纤维化,纤维化性肺疾病,间质性肺炎,间质性肺疾病,纤维化性肺病,间质性肺病,巨细胞病毒,EB病毒,胃食管反流,端粒酶基因,干咳,劳力性呼吸困难,杵状指,爆裂音,发绀,肺动脉高压,肺心病,右心功能不全,杵状趾,血清标记物,类风湿因子,抗环瓜氨酸,中性粒细胞,抗核抗体谱,肺功能检查,通气功能,容量测定,弥散功能,用力肺活量,胸部HRCT,病理诊断,外科肺活检,成纤维母细胞灶,平滑肌增生,室内外环境暴露,结缔组织病,药物性肺损害,网格状阴影,蜂窝样改变,支气管扩张,磨玻璃样影,微结节,散在囊状病变,弥漫性马赛克征,气体陷闭,肺基底部,支气管血管束,肺叶实变,肺段实变,过敏性肺泡炎,尘肺,类风湿关节炎,多血管炎,戒烟,氧疗,低氧血症,呼吸支持,无创呼吸机,肺栓塞,缺血性心肌病,不稳定心绞痛,急性心肌梗塞,机械通气,肺康复锻炼,肌肉训练,肺移植,药物治疗,吡非尼酮,尼达尼布,光敏性皮疹,胃肠道反应,肝功能损害,腹泻,抗胃食管反流,质子泵抑制剂,组胺受体拮抗剂,N-乙酰半胱氨酸,华法林,糖皮质激素,辅助呼吸,免疫抑制剂,支气管肺泡灌洗,病友,关爱中心,互助家园,IPF |
| Systemic Sclerosis | 系统性硬化症,自身免疫病,皮肤纤维化,内脏器官纤维化,微血管病变,局限性皮肤型SSc,皮肤硬化,弥漫性皮肤型SSc,重叠综合征,systemic sclerosis,硬皮病,遗传易感性,表观遗传学因素,HLA-DBQ1-0501,抗着丝点抗体,HLA-DRB1-1104,DPB1-1301,抗拓扑异构酶抗体,STAT4,BANK1,化学物质,内分泌因素,免疫反应,血管病变,成纤维细胞活化,雷诺现象,胃食管反流,皮肤肿胀,硬化,皮肤色素改变,色素沉着,色素脱失,四肢远端,面颈部,四肢近端,肘关节近端,膝关节近端,躯干受累,关节痛,肌痛,指端溃疡,毛细血管扩张,内脏器官受累,呼吸系统,肺间质纤维化,肺动脉高压,消化系统,胃窦血管扩张症,腹痛,腹泻,便秘,大便失禁,肾危象,急性肾功能不全,高血压,心脏受累,心脏受累,心脏传导系统异常,心肌受累,神经系统,外周神经系统受累,三叉神经病变,周围神经病变,中枢神经受累,CREST综合征,钙质沉积,食管功能障碍,指硬化,趾硬化,毛细血管扩张,Rodnan皮肤评分,皮肤增厚,抗核抗体,微出血,抗拓扑异构酶Ⅰ抗体,SCL-70,抗RNA聚合酶III抗体,肺间质病变,肿瘤,肺功能检查,肺的容量,通气功能,弥散功能,限制性通气功能障碍,胸部高分辨CT,毛细血管密度,非特异性间质性肺炎,毛细血管管腔,胸膜,网格影,磨玻璃影,牵拉性支气管扩张,蜂窝肺,上消化道造影,食道运动障碍,食道扩张,超声心动图,心脏病变,右心导管检查,心肌核磁共振,甲褶微循环,手指肿胀,指端凹陷型瘢痕,皮肤纤维化,皮下组织纤维化,硬肿病,嗜酸性筋膜炎,浸润性病变,化学物质,毒物,硬皮病样综合征,周围血管病变,血管炎,系统性红斑狼疮,混合性结缔组织病,炎性肌病,类风湿关节炎,甲氨蝶呤,吗替麦考酚酯,激素,泼尼松,环磷酰胺,托珠单抗,硫唑嘌呤,器官衰竭,造血干细胞移植,二氢吡啶类,钙离子拮抗剂,硝苯地平,5-磷酸二酯酶抑制剂,静脉前列环素类药物,内皮素受体拮抗剂,波生坦,西地那非,他达拉非,安立生坦,马西生坦,联合药物治疗,静脉依前列醇,硬皮病肾危象,血管紧张素转换酶抑制剂,ACEIs,糖皮质激素,血压,肾功能,质子泵抑制剂,胃肠动力障碍,小肠细菌,抗生素,营养支持,免疫抑制治疗,心功能不全,双磷酸盐类药物,螯合剂,手术切除,病友,关爱中心,互助家园,Scl-70,Scl70,Scl 70,SSc |
| Amyotrophic Lateral Sclerosis | 大脑皮质,脑干,脊髓运动神经,神经系统变性,进行性球麻痹,连枷臂,连枷腿,肌萎缩,侧索硬化,肌无力,肌束颤动,锥体束,RNA加工异常,谷氨酸兴奋性毒性,细胞骨架排列紊乱,线粒体功能障碍,病毒感染,细胞凋亡,生长因子异常,炎症反应,运动神经元同时受累,认知功能障碍,额颞叶痴呆,呼吸衰竭,体格检查,运动神经元受累,肌肉无力,腱反射亢进,肌张力,阵挛,痉挛,吸吮反射,咽反射,下颌反射,掌颌反射,延髓麻痹,四肢腱反射,霍夫曼征,下肢病理征,腹壁反射,电生理检查,肌电图,神经传导测定,运动神经元病变,远端运动潜伏期,神经传导速度,传导阻滞,异常波形离散,复合肌肉动作电位波幅,在嵌压性周围神经病,F 波测定,进行性失神经,慢性失神经,正锐波,束颤电位,发放频率升高,募集相减少,多相波增多,发放不稳定,胸锁乳突肌,舌肌,面肌,咬肌,脊旁肌,腹直肌,神经源性,运动诱发电位,经颅磁刺激,运动传导时间,颈神经,结构性病变,脊髓病,SOD1基因,ANG基因,VAPB基因,VCP基因,SQSTM1基因,TARDBP基因,DCTN1基因,DAO基因,SETX基因,FUS基因,C9ORF72基因,ATXN2基因,OPTN基因,SCFD1基因,NEK1基因,C21ORF2基因,感觉症状,括约肌障碍,视觉障碍,自主神经功能,基底神经节功能障碍,阿尔茨海默型痴呆,压迫综合征,获得性酶缺陷,自身免疫性综合征,内分泌异常,多灶性运动神经病,平山病,颈椎病,腰椎病,成人脊髓性,肯尼迪病,痉挛性截瘫,苯并噻唑,电压门控钠通道,谷氨酸释放,谷氨酸摄取,谷氨酸受体,疲乏,恶心,肝转氨酶,呼吸机,依达拉奉,流涎,吞咽困难,脱水,体重下降,呛咳,胃造瘘术,鼻胃管,神经内科,呼吸内科,神经科,消化内科,心理科,康复科,营养科,抑郁,焦虑,失眠,护理,病友,关爱中心,互助家园,ALS,SOD1,TDP-43,TDP43,TDP 43,FUS,C9ORF72,TARDBP |
| Hepatolenticular Degeneration(Wilson Disease) | 肝豆状核变性,铜离子转运障碍,铜离子排泄障碍,脏器蓄积,ATP7B基因,酶功能障碍,肝脏铜蓝蛋白,多脏器损害,铜转运蛋白,P型ATP酶,胆道铜,肝脏沉积,肝细胞坏死,肝脏受累,神经系统受累,转氨酶,慢性肝炎,肝硬化,肝功能衰竭,运动功能障碍,震颤,共济失调,舞蹈症,自主运动障碍,肌张力障碍,面具脸,四肢僵硬,步态异常,精神行为异常,溶血性贫血,脾功能亢进,凝血功能异常,血液三系下降,肝功能检查,肾脏检查,肝酶升高,胆红素升高,胆汁酸升高,凝血时间延长,低蛋白血症,血尿,蛋白尿,肝实质光点增粗,肝脏增大,结节状改变,轻度脂肪变性,头颅核磁,脑沟增宽,脑室扩大,肝细胞内糖原化,局灶性肝细胞坏死,头颅核磁,累及基底节,纤维化,K-F环阳性,角膜色素环,角膜后弹力层,慢性病毒性感染,自身免疫性肝炎,HFE相关,药物性肝损,硬化性胆管炎,血色素沉着症,抗胰蛋白缺乏症,酒精性肝病,铜蓝蛋白降低,24小时尿铜增高,铜螯合剂,青霉胺,骨髓抑制,肾损害,皮肤损害,药物性狼疮,曲恩汀,青霉胺不耐受,二巯丁二酸,血清氨基转移酶,二巯丙磺酸钠,金属硫蛋白诱导剂,锌剂,硫酸锌,葡萄糖酸锌,醋酸锌,胃肠道刺激症状,无症状血清脂肪酶,淀粉酶升高,缺铁性贫血,肝移植,暴发性肝衰竭,失代偿性肝硬化,尿铜,可逆性缺铁性贫血,MLPA分析,病友,关爱中心,互助家园,ATP7B |
| Autoimmune Encephalitis | 自身免疫机制介导,脑炎,抗神经抗体,抗NMDA受体,肿瘤,脑脊髓炎,Bickerstaff脑干,bickerstaff脑干,细胞免疫反应介导,中枢神经系统损伤,细胞表面蛋白,抗NMDAR抗体,neurexin-3α,神经元坏死,前驱感染事件,基底节脑炎,抗细胞内抗原抗体,抗细胞表面抗体,边缘性脑炎,小细胞肺癌,精原细胞瘤,胸腺瘤,乳腺癌,卵巢畸胎瘤,Morvan综合征,腹泻,睡眠障碍,淋巴瘤,霍奇金淋巴瘤,僵人综合征,亚急性小脑变性,感觉神经元神经病,肌强直,阵挛,morvan综合征,精神行为异常,认知障碍,记忆力下降,抗IgLON5抗体,言语障碍,运动障碍,不自主运动,意识水平下降,自主神经功能障碍,CNS局灶性损害,神经肌肉接头受累,弥漫性脑炎,癫痫,近记忆力障碍,莫旺综合征,共济失调,抗DPPX抗体,脑脊液异常,淋巴细胞性炎症,白细胞增多,寡克隆区,电生理异常,FLAIR异常信号,非特异性白质改变,卒中,高代谢改变,EEG异常,慢波节律,畸胎瘤,亚急性起病,边缘系统症状,近事记忆减退,多灶性脑损,间脑受累,精神障碍,自身抗体阳性,间接免疫荧光法,下丘脑受累,遗传性小脑变性,神经梅毒,中枢神经系统感染,Creutzfeldt-Jakob 病,中毒性脑病,Wernicke脑病,肝性脑病,肺性脑病,肾性脑病,青霉素类,喹诺酮类等抗生素,多系统萎缩,放射性脑病,肿瘤性疾病颅内原发肿瘤,大脑胶质瘤病,原发CNS淋巴瘤,颅内转移瘤,变性病线粒体脑病,线粒体脑肌病,甲基丙二酸血症,肾上腺脑白质营养不良,路易体痴呆,免疫治疗,糖皮质激素,静脉免疫球蛋白,利妥昔单抗,静脉环磷酰胺,吗替麦考酚酯,硫唑嘌呤,急性起病,抗利尿激素分泌不当,顽固性低钠血症,杏仁体肥大,窦性心动过速,心动过缓,泌涎增多,中枢性低通气低血压,中枢性发热,复视,FLAIR高信号,皮质点片状,双侧枕叶代谢明显减低,可见代谢异常,异常δ刷,卵巢超声,和盆腔CT,卵巢微小畸胎瘤,基底节区异常信号,昏迷,肌张力障碍,双侧颞叶内侧异常信号,FLAIR相敏感,病友,关爱中心,互助家园,NMDAR,IgLON5,DPPX,AMPAR,GFAP,LGI1,GABABR |
| Hemophilia | 凝血因子,基因缺陷,基因倒位突变,重现性突变,关节出血,肌肉出血,胃肠道出血,中枢神经出血,关节畸形,假肿瘤,延迟性出血,凝血活酶,APTT延长,酶谱分析法,Southern印迹法,聚合酶链反应,DNA测序,PCR法,瘀伤,自发性出血,过量出血,出血家族史,羊膜穿刺术,绒毛膜取样,皮肤出血,黏膜出血,鼻出血,月经过多,阿司匹林,非甾体类解热镇痛药物,旁路制剂,替代治疗,凝血酶原复合物,过敏,肾病综合征,关节炎滑膜炎,血友病假肿瘤,囊性包裹性血肿,丙型病毒肝炎,乙型病毒感染,人类免疫缺陷病毒,艾美赛珠单抗,组织因子通路抑制剂,人源化单克隆抗体,RNAi疗法,基因治疗,自发瘀伤,抗磷脂抗体,凝血因子抑制物,病友,关爱中心,互助家园 |
| Neuromyelitis Optica | 脊髓受累,炎性脱髓鞘疾病,Devic病,中枢神经系统受累,devic病,特异性水通道蛋白,AQP4-IgG,炎症介质释放,炎症反应浸润,星形胶质细胞足突,细胞毒,第三和第四脑室周围,胼胝体,脱髓鞘病灶,少突胶质细胞损伤,髓鞘脱失,室管膜,延髓最后区,亚急性起病,视神经炎,急性脊髓炎,极后区综合征,急性脑干综合征,症状性睡眠发作,急性间脑临床综合征,脑部病变,高复发,高致残性疾病,复发性病程,神经功能残疾,急性起病, NMOSD,实验室检查,血清AQP4-IgG,特异性生物学标记物,血清标本,脑脊液,免疫抑制治疗,脑脊液指标,细胞数,寡克隆区,多发性硬化,脊髓长节段病灶,椎体节段,轴位,灰质,白质,颈髓,胸髓,急性期病灶,脊髓肿胀,增强扫描,空洞样改变,髓下部,萎缩,受累视神经肿胀增粗,皮质下区,三脑室,四脑室,大脑脚,视觉诱发电位,P100 潜伏期,OCT 检查,视网膜神经纤维层,核心临床症候,影像特征,诊断标准,视神经脊髓炎谱系疾病,长节段横贯性脊髓炎,空间播散性,急性视神经炎,脑MRI,非特异性白质,视交叉,最后区综合征,髓背侧,最后区病灶,伴发室管膜,脑干病变,中枢神经系统脱髓鞘病,急性播散性脑脊髓炎,假瘤型脱髓鞘病,血管性疾病,缺血性视神经病,脊髓血管畸形,亚急性坏死性脊髓病,布氏杆菌感染,热带痉挛性截瘫,中毒性视神经病,亚急性联合变性,肝性脊髓病,Wernicke 脑病,缺血缺氧性脑病,遗传性疾病,Leber视神经病,遗传性痉挛性截瘫,肾上腺脑白质营养不良,脊髓胶质瘤,室管膜瘤,脊髓副肿瘤综合征,颅底畸形,脊髓压迫症,风湿免疫性疾病,干燥综合征,白塞病,系统性红斑狼疮,结节病,系统性血管炎,脑脊液细胞,致盲率,复发-缓解型,脑脊液寡克隆区,IgG指数,脊髓MRI,脑MRI,皮质下,导水管周围斑片状,片状高信号病灶,侧脑室旁白质圆 形,特异性治疗,急性期,残疾程度,糖皮质类固醇激素,静脉滴注免疫球蛋白,甲泼尼龙冲击疗法,血浆置换,激素,免疫抑制剂,脊髓侵袭,缓解期,复发,延缓残疾进展,硫唑嘌呤,吗替麦考酚酯,甲氨蝶呤,利妥昔单抗,环磷酰胺,米托蒽醌,他克莫司,静脉滴注免疫球蛋白,肢体、语言、吞咽等功能障碍,功能康复锻炼,遗传,婚姻,IVIG,并发症,痛性痉挛,呃逆,慢性疼痛,膀胱直肠功能障碍,性功能障碍,病友,关爱中心,互助家园,NMOSD,NMO,AQP4 |
| Retinoblastoma | 眼内恶性肿瘤,视网膜干细胞,视锥细胞前体细胞,松果体瘤,三侧性RB患者,双眼RB,遗传型RB,RB1基因,视网膜细胞,染色体13q14,外显子,RB蛋白,细胞周期调控,抑制细胞异常增殖,胚胎期RB1突变,缺少RB蛋白,细胞增生,新生儿RB,白瞳症,胃肠道癌,黄白色反光,后极部,肿瘤,患眼,视力低下,失用性斜视,眼内期,青光眼期,眼外期,全身转移期,恶性特征,视网膜细胞瘤,眼内肿块,椭圆形拱状肿物,玻璃体隆起,沿脉络膜扁平生长,视网膜血管,渗出性视网膜脱离,瘤组织,穿破视网膜,雪球状漂浮,沉积于前房,假性前房积脓,双侧性RB患儿,颅内肿瘤,松果体母细胞瘤,异位性颅内,RB基因突变,三侧性RB,单侧性RB患儿,生殖细胞性,骨肉瘤,软组织肉瘤,黑色素瘤,恶性肿瘤,乳腺癌,B超,玻璃体内弱回声,中强回声光团,眼底光带,强回声钙化斑,眼底光带不均匀增厚,视网膜脱离,彩色多普勒超声成像,红蓝伴行,CT成像,眼内高密度肿块,肿块内钙化灶,视神经增粗,视神经孔扩大,眶骨受侵,非肿瘤病变,Coats病,MRI成像,类圆形结节样病灶,显著高信号,视神经和眼外受累,异位性颅内RB,生殖细胞突变,大片段插入,稍低及稍高混杂,增强扫描,全外显子测序,全基因组测序,外周血,术中冰冻切片,术后肿块病检,眼内穿刺检查,播散转移,双眼发病,肿瘤细胞轮辐状排列,视网膜内白色小球,胞浆比高,HE染色呈蓝色,TNMH分期,眼内肿瘤,新生血管形成,网膜下积液,网膜下播散,眼球痨,玻璃体播散,肿瘤浸润脉络膜,无菌性眼眶蜂窝织炎,眼内压升高,牛眼症,RB1体细胞突变,前房积血,玻璃体大量出血,嵌合体风险,脉络膜浸润,视神经乳头,肿瘤浸润虹膜基质,累及巩膜层,浸润视神经,浸润巩膜全层,转移性眼内炎,高热后发病,玻璃体内大量渗出,前房积脓,全身异常,小头,微血管瘤,小角膜,低体重,早产史,高浓度吸氧史,增生性病变,牵拉性视网膜脱离,增生病变收缩,玻璃体持续增生症,视网膜病变,Norrie氏病,不典型RB,睫状突牵引,静脉化学治疗,动脉化学治疗,激光治疗,冷冻治疗,鞘内注射化学治疗,遗传性RB,眼外RB,长春新碱,依托泊苷,卡铂,恶心,呕吐,发热,血细胞减少,听力损伤,肾功能损伤,第二恶性肿瘤,美法仑,托普替康,缩小肿瘤体积,后极部病灶,周边部病灶,控制残留病灶,摘除眼球,眼球摘除术,最大长度切除视神经,全身化学治疗,外放射治疗,结膜水肿,眶内容物摘除术,淋巴结清扫手术,散瞳眼底检查,裂隙灯前节检查,眼球突出,玻璃体腔白色团块混浊,虹膜表面灰白结节,眼压增高,瞳孔散大,病友,关爱中心,互助家园,RB-1,RB1,RB 1 |
| Retinitis Pigmentosa | 视网膜,光感受器,色素上皮变性,遗传异质性,细胞凋亡,致盲眼病,综合征性RP,基因位点,眼部异常,遗传综合征,Usher综合征,Bardet-Biedl综合征,Alström综合征,Refsum综合征,Cockayne综合征,Hunter综合征,双基因遗传RP,线粒体遗传RP,光信号转导,维生素A循环,视黄醇,基因转录,RNA剪切,胞内物质运输,ADRP基因,ARRP基因,XLRP基因,生物学通路,RHO基因,RPGR基因,细胞间互相作用,光感受器结构,青少年时期起病,视杆细胞,视锥细胞,色素上皮细胞,夜盲,首发症状,视力下降,周边视野缺损,中心视力下降,法定盲人,视功能损伤,视力预后,眼底表现,中周部眼底照相,早期毯层样,扫描激光眼底照相,广域眼底照相,眼底改变,骨细胞样,色素沉积,视盘蜡黄,Coat’s 样,视盘玻璃膜疣,脱色素改变,广泛变性,黄斑囊样变性,后囊下白内障,玻璃体尘样颗粒,高荧光,椭圆体带消失,RPE 层变薄,中心凹下,椭圆体带,低荧光,RPE萎缩区,RPE病变,熄灭型,视野,代偿功能,视野缺损,管状视野,自发荧光,眼底改变者,ERG检查,暗处视力差,ERG5 项,暗视反应,不规则色素团块,骨细胞样色素,椒盐样色素,灰白色素,锥杆细胞营养不良,损害视锥细胞,视杆细胞损害,累及黄斑区,视网膜色素变性,视锥细胞损害,视力减退,色觉异常,明视反应损害,Leber先天黑矇,脉络膜血管萎缩,视功能损害严重,盲童,视力异常,眼球震颤,瞳孔反射迟钝,畏光,ERG呈熄灭型,早发视网膜变性,无脉络膜症,露出巩膜白色反光,X连锁RP,X 连锁隐性遗传,眼底赤道部,脉络膜萎缩,色素脱失,脉络膜毛细血管层,RPE层萎缩范围扩大,脉络膜大血管,RP发病机制,神经保护,基因治疗,干细胞治疗,人工视网膜,注射眼,对侧眼,rds小鼠,MERTK相关,rAAV5载体,RP3基因,视网膜下腔,视锥感光功能,保护外丛状层,双基因治疗策略,CHM基因突变检测,siRNA,抑制RDS,AAV载体,基因替代治疗,视网膜下腔注射治疗,裂隙灯前节检查,全身发育性异常,视神经色淡,病友,关爱中心,互助家园,RP,CYP4V2,RHO,USH 2A,RPGR,EYS |
| Progressive Muscular Dystrophy | 骨骼肌,异质性,胃肠道受累,起病时间,进展速度,受累范围,X连锁隐性遗传,肌营养不良,Becker型,Emery-Dreifuss型,Duchenne型,DMD基因,面肩肱型,4q亚端粒区,巨卫星串联,重复序列,DUX4基因,SYNE2基因,SMCHD1基因,TMEM43基因,EMD基因,FHL1基因,LMNA基因,SYNE1基因,肢带型,眼咽型,无力萎缩,PABPN1基因,INPP5K基因,MYOT基因,CAV3基因,DNAJB6基因,DES基因,TNPO3基因,HNRNPDL基因,糖基化相关,CAPN3基因,DYSF基因,SGCG基因,SGCA基因,SGCB基因,SGCD基因,TCAP基因,TRIM32基因,TTN基因,ANO5基因,PLEC基因,TRAPPC11基因,TOR1AIP1基因,LIMS2基因,BVES基因,POGLUT1基因,B4GAT1基因,Dystroglycan,POMT1基因,POMT2基因,POMGNT1基因,FKTN基因,FKRP基因,LARGE1基因,ISPD基因,POMGNT2基因,DAG1基因,TMEM5基因,B3GALNT2基因,POMK基因,B3GNT1基因,GMPPB基因,基因缺陷,LAMA2基因,COL6A1基因,COL6A2基因,COL6A3基因,COL12A1基因,SELENON基因,ITGA7基因,CHKB基因,TRIP4基因,维持肌肉细胞,伸缩过程,肌膜完整性,结构蛋白,编码基因,致病缺陷,致功能异常,肌肉进行性破坏,儿童期起病,假肥大体征,运动发育轻度迟滞,影响肢体运动功能,步态异常,上肢活动受限,自然病程,丧失行走能力,肢体无力,舌肌假肥大,呼吸肌无力,扩张性心肌病,呼吸衰竭,心功能衰竭死亡,双腓肠肌假肥大,双前臂假肥大,Gower’s征阳性,腰椎前凸,相对良性表型,血清学检测,肌酸激酶,高通量测序,乳酸脱氢酶,拷贝数异常,羟丁酸脱氢酶,大片段缺失,谷草转氨酶,生物信息学分析,谷丙转氨酶,多重连接探针扩增技术,肌红蛋白,微小突变,肌细胞损害,肌电图,肌肉疾病,轻型BMD,肌酶升高,针极肌电图,神经传导速度检查,肌源性损害,肌肉MR,肌肉病变发展,炎性水肿,脂肪替代,受累肌群分布特点,肌肉活检,肌营养不良样,形态学改变,免疫组化,免疫荧光染色,炎性肌病,代谢性肌病,基因检测,跟腱挛缩,血肌酶谱明显升高,神经肌肉病,运动神经元病,破碎红纤维,肌肉病,脂滴增多,其他类型肌营养不良,肌酶明显升高,肌聚糖蛋白病,线粒体病,波动性病程,血肌酶升高,肌细胞内糖原沉积,4号染色体亚端粒区,Southern blot,光学图谱技术,Bionano,面肩肱型肌营养不良,脊肌萎缩症,MHC-Ⅰ表达,肢体近端无力萎缩,血肌酶轻度升高,广泛神经源性损害,多发性肌炎,皮肌炎,包涵体肌炎,合并大量自发电位,活跃期表现,炎性细胞浸润,肌纤维膜,LGMD2B,糖原累积性肌病,脂质沉积性肌病,延缓疾病进展,延长生存期,规范口服激素治疗,泼尼松,泼尼松龙,每日疗法,周末疗法,间断疗法,甲泼尼龙,地夫可特,康复治疗,关节挛缩,姿势异常,肌力不足,站斜板,足部矫形支具,外科手术治疗,脊柱侧弯,外科评估,多器官系统受累,骨质疏松,二磷酸盐,心功能下降,抗心衰药物,呼吸功能下降,基因治疗,细胞治疗,外显子51跳跃药物,AAV载体,外源截短,基因编辑,干细胞治疗,eteplirsen,渐进性肢体无力萎缩,肢体近远端肌力,双下肢肌肉MR,病友,关爱中心,互助家园,DMD,SMCHD1 |
| Hereditary Epidermolysis Bullosa | 大疱性表皮松解症,半桥粒,表皮角蛋白,表皮松解症,炎症细胞浸润,皮肤脆性增加,表皮内疱,表皮下疱,真表皮间锚定蛋白,KRT5基因,KRT14基因,角蛋白,桥粒斑蛋白,表皮基底膜,锚原纤维,锚丝结构缺陷,COL7A1基因,胶原,水疱,大疱,口腔黏膜,恶性黑色素瘤,吞咽困难,声音嘶哑,色素异常,秃发,掌跖角化过度,牙釉质缺陷,甲增厚,甲营养不良,反复瘢痕,指端残毁,贫血,骨质疏松,肌肉萎缩,心肌病,耳聋,基底细胞癌,鳞状细胞癌,泌尿生殖器损害,黏膜受累,牙齿牙釉质,萎缩性秃发,口腔黏膜病变,前鼻孔狭窄,幽门梗阻,进行性感知耳聋,掌跖角化,掌跖多汗,粟丘疹,疱疹样型,斑驳色素型,肌营养不良,侏儒,爪形手,指骨萎缩,假性并指,遗传营养不良,恶性黑素瘤,Herlitiz型,甲缺失,牙齿变形,支气管损害,呼吸窘迫,骨骼肌肉变形,胃肠道损害,皮损普通组织病理,皮肤免疫荧光病理,盐裂实验,皮肤组织病理,免疫荧光,透射电镜,基因检测,获得性大疱性表皮松解症,Ⅶ型胶原,天疱疮,红斑,张力性大疱,瘙痒剧烈,卟啉病,瘢痕,色素沉着,多毛,硬皮症,糖皮质激素,预防创面,肢体残毁,活动受限,食道狭窄,皮肤活检,炎症浸润,免疫荧光阴性,机械损伤,创面感染,病友,关爱中心,互助家园, KRT5, KRT14,PLEC1,DSP,COL7A1,LAMA3,LAMB3,LAMC2,COL17A1,ITGB4,ITGA6 |
| Paroxysmal Nocturnal Hemoglobinuria | PNH,PIG-A,PIGA,PIG A |
