# Supplementary material for: Estimating Rare Disease Incidences With Large-scale Internet Search Data: Development and Evaluation of a Two-step Machine Learning Method
Source: JMIR Infodemiology. 2023 Apr 28;3:e42721. doi: 10.2196/42721 (PMC10182453; doi:10.2196/42721)
Supplement: Multimedia Appendix 4 [file infodemiology_v3i1e42721_app4.docx]

## Multimedia Appendix 4: Representative Sessions with different intents

To show the performance of session intent prediction method in detail, four representative sessions with different intents are shown in Table 1. All of them were correctly predicted with the intent prediction model. The first and second sessions were RD-concerned and News-concerned sessions, respectively. The third session was searching for another scenario (i.e., drawing) but accidentally contained one query (progressive muscular dystrophy) on an RD. And the fourth session was probability asked by a medical student doing his/her assignments.

Four representative sessions with different intents. The types of URL domains are marked after the URLs. (Ns: News, Med: Medical knowledge, Wiki: General Wiki, Edu: Education)

| **Session Intent** | **Query** | **Clicked Doc. Pos.** | **Clicked Doc. URL Domain** |
| --- | --- | --- | --- |
| RD-concerned | Advances in gene therapy for hemophilia | 13, 7, 8, 2 | zhihu.com(Med),  doc88.com (Wiki),  360doc.cn (Wiki),  120ask.com (Med) |
|  | Can gene therapy cure hemophilia? | 2, 29 | wk.baidu.com(Wiki), xywy.com(Med) |
|  | How much is cryoprecipitate? | 5, 2, 8 | wk.baidu.com(Wiki), xywy.com(Med), med66.com(Med) |
| News-concerned | What is ALS? | 2 | pb.sogou.com (Wiki) |
|  | Female PhD at Peking University | - | - |
|  | ALS, female PhD at Peking University | 1 | news.ifeng.com (Ns) |
| Others | How to draw a picture of muscle atrophy | 1 | blog.sina.com (Wiki) |
|  | Progressive muscular dystrophy | - | - |
|  | How to draw a picture of atrophy | - | - |
| Others | Which is true about the treatment of primary pulmonary hypertension | 2 | xinghengedu.com (Edu), baike.com (Wiki) |
|  | Major treatments of brain resuscitation | 1 | wk.baidu.com (Wiki) |
|  | In ulcerative colitis complicated with toxic megacolon, which part is the most severe | 1 | shangxueba.com (Edu) |
