# Supplementary material for: Estimating Rare Disease Incidences With Large-scale Internet Search Data: Development and Evaluation of a Two-step Machine Learning Method
Source: JMIR Infodemiology. 2023 Apr 28;3:e42721. doi: 10.2196/42721 (PMC10182453; doi:10.2196/42721)

## Multimedia Appendix 5: Comparison between Session Input and Query Input for Estimation

To show details of the differences between session and query as input, we display the average Relative Error Rate (RER) of each disease in each region on the test set (i.e., four seasons in 2019) in Figure 5, where darker colors demonstrate larger errors. Qualitatively, the left part of Figure 5 is much shallower than the right, indicating the error was generally smaller on distinct diseases and regions with Session input.

For quantized comparison, the RERs of different diseases averaged on the test set are shown in Figure 6. Session input shows lower RER on most RDs, illustrating consistently better performance across diseases. The exception, Disease 8 (Hemophilia), showed a slightly opposite trend. It is because Hemophilia was well known to the public and easily confused with several common blood disorders in Chinese (such as leukemia and anemia). Hence, users’ behavior on the search engine contained more noise when more information about the search process, i.e., the session context, was considered. As for estimation error values of different RDs, RERs on most RDs are fewer than 0.5. Considering the sparsity of RD cases, the performance is encouraging for RDs incidence estimation.

Average Relative Error Rate (RER) of each disease in each region on the test set (i.e., average of four seasons in 2019).


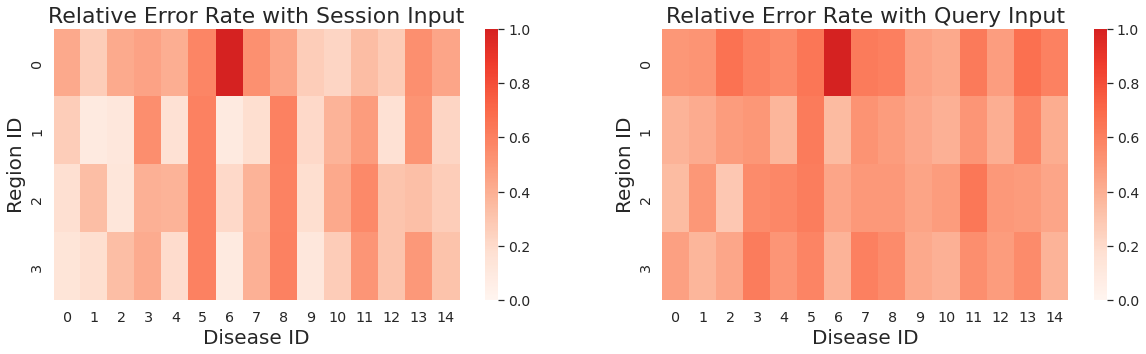


Average Relative Error Rate (RER) of each disease on the test set (i.e., average of four regions in four seasons in 2019).


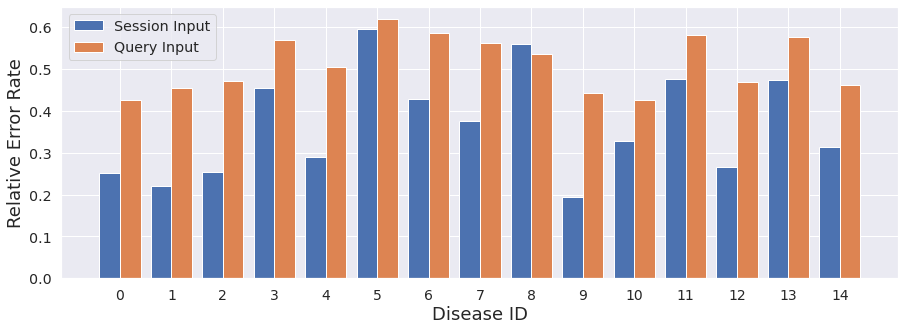

Supplement: Multimedia Appendix 5 [file infodemiology_v3i1e42721_app5.docx]
